# Striatal Infarction Elicits Secondary Extrafocal MRI Changes in Ipsilateral Substantia Nigra

**DOI:** 10.1371/journal.pone.0136483

**Published:** 2015-09-01

**Authors:** Benjamin Winter, Peter Brunecker, Jochen B. Fiebach, Gerhard Jan Jungehulsing, Golo Kronenberg, Matthias Endres

**Affiliations:** 1 Center for Stroke Research Berlin (CSB), Charité-Universitätsmedizin Berlin, Berlin, Charitéplatz 1,10117, Berlin, Germany; 2 Department of Neurology, Charité-Universitätsmedizin Berlin, Charitéplatz 1, 10117, Berlin, Germany; 3 Department of Neurology, Jüdisches Krankenhaus Berlin, Heinz-Galinski-Strasse 1, 13347, Berlin, Germany; 4 Department of Psychiatry, Charité-Universitätsmedizin Berlin, Charitéplatz 1, 10117, Berlin, Germany; 5 Max-Delbrück Center and Charité Medical Faculty, Experimental and Clinical Research Center, Lindenbergerweg 80, 13125, Berlin, Germany; 6 Excellence Cluster Neurocure, Charité-Universitätsmedizin Berlin, Charitéplatz 1, 10117, Berlin, Germany; 7 German Center for Neurodegenerative Diseases (DZNE), Ludwig-Erhard-Allee, 53175, Bonn, Germany; 8 German Centre for Cardiovascular Research (DZHK), Oudenarder Straße 16, 13347, Berlin, Germany; Julius-Maximilians-Universität Würzburg, GERMANY

## Abstract

Focal ischemia may induce pathological alterations in brain areas distant from the primary lesion. In animal models, exofocal neuron death in the ipsilateral midbrain has been described after occlusion of the middle cerebral artery (MCA). Using sequential magnetic resonance imaging (T2- and diffusion-weighted) at 3 Tesla, we investigated acute ischemic stroke patients on days 1, 2, 6, 8, and 10 after stroke onset. Sixteen consecutive patients who had suffered a stroke involving the caudate nucleus and/or putamen of either hemisphere were recruited into the study. Four additional patients with strokes sparing the caudate nucleus and putamen but encompassing at least one-third of the MCA territory served as controls. Ischemic lesions involving striatal structures resulted in hyperintense lesions in ipsilateral midbrain that emerged between days 6 and 10 after stroke and were not present on the initial scans. In contrast, none of the control stroke patients developed secondary midbrain lesions. Hyperintense lesions in the pyramidal tract or the brain stem caused by degeneration of the corticospinal tract could be clearly distinguished from these secondary midbrain gray matter lesions and were detectable from day 2 after ischemia. Co-registration of high-resolution images with a digitized anatomic atlas revealed localization of secondary lesions primarily in the substantia nigra pars compacta. Apparent diffusion coefficient (ADC) values in the secondary lesions showed a delayed sharp decline through day 10. Normalization of ADC values was observed at late measurements. Taken together, our study demonstrates that striatal infarction elicits delayed degenerative changes in ipsilateral substantia nigra pars compacta.

## Introduction

Midbrain changes associated with the loss of basal ganglia neurons have been described in experimental models of brain ischemia in rats [1–6], mice [7, 8], gerbils [9], and cynomolgus monkeys [10]. Magnetic resonance imaging (MRI) studies in experimental rodents demonstrate transient exofocal signal changes in ipsilateral midbrain. In particular, apparent diffusion coefficient (ADC) values show a significant delayed decrease and T2 values show a significant delayed increase in the ipsilateral midbrain after occlusion of the middle cerebral artery [8, 11, 12]. On the histological level, these subacute MRI changes after experimental brain ischemia are associated with alterations such as cellular swelling and changes in neuronal morphology [11, 13].

The anecdotal histopathological reports of human autopsy brain tissue published so far indicate nerve cell loss in the ipsilateral substantia nigra during the chronic phase after a large infarction of the basal ganglia [14, 15]. Correspondingly, several MRI investigations of patients with a stroke in the striatum also revealed a T2-weighted hyperintensity appearing as a subacute exofocal alteration in the ipsilateral midbrain [16–19]. Here, we present a systematic prospective study of a series of 16 consecutive patients with striatal infarction admitted to our stroke unit between January 2009 and May 2010. Four additional patients whose ischemic lesions spared the striatum served as controls. Sequential high-resolution anatomical MRI at 3 Tesla was used to delineate the emergence and the precise neuroanatomic localization of secondary MRI changes in the midbrain.

## Methods

### Patients and Controls

Data presented here were collected within the framework of a larger project designated the "1000Plus study”, a prospective, single-center observational investigation [20, 21]. Study details are posted at www.clinicaltrials.gov (NCT00715533). Based on the initial MRI assessment (see below), 16 consecutive patients who had suffered an acute ischemic stroke involving the caudate nucleus and/or putamen of either hemisphere were recruited into the study (‘striatal stroke patients’). Four additional patients who had suffered an acute ischemic stroke sparing caudate nucleus and putamen but typically encompassing at least 1/3 of the middle cerebral artery (MCA) cortical territory served as controls (‘control stroke patients’). Exclusion criteria included severe aphasia or inability to provide informed consent, hemorrhagic stroke, severe brain damage before admission, moderate to severe dementia (MMSE < 18) as well as contraindications to MRI. The protocol had been approved by the local ethics committee and written informed consent was obtained prior to the study from each participant according to the Declaration of Helsinki.

### Image Acquisition

All MRI scans were acquired on a 3 T whole-body scanner (TIM Trio; Siemens AG, Erlangen, Germany) dedicated entirely to clinical research. The baseline scan was obtained within 24 hours of stroke onset (day 1). Follow-up scans were performed on days 2, 6, 8, and 10. In some patients, additional later scans were performed as shown in Table 1. On days 1 and 2, the MRI protocol for this investigation included the following transversely-oriented sequences with a consistent field-of-view of 230 mm: T2*-weighted imaging (2D-FLASH; TA = 73 s; TE = 20 ms; TR = 620 ms; flip angle = 20°; matrix = 256 x 205; 25 slices with 5 mm thickness and 5.5 mm spacing); diffusion-weighted imaging (DWI) (SE-EPI; TA = 131 s; TE = 93 ms; TR = 7600 ms; matrix = 192 x 192, 50 slices with in-plane resolution of 1.2 mm, thickness of 2.5 mm and no interslice gap; 6 directions; b-values = 0 and 1000 s/mm^2^). On days 6, 8, and 10, the protocol included DWI as described above as well as coronal T2-weighted imaging (TSE; TA = 157 s; TE = 79 ms; TR = 5540 ms; matrix = 384 x 307; 30 slices with in-plane resolution of 0.6 mm, thickness of 3 mm and spacing of 3.3 mm).

**Table 1:** MRI findings in striatal stroke patients and control stroke patients.

Patient no.	Putamen	Caudate Ncl.	Pallidum	<1/3 MCA cortex	>1/3 MCA cortex	Cm^3^	Day 6	Day 8	Day 10	Day 12	Day 72–144
1	X	X		X		0.8	–	+	+		
2	X	X			X	126.1	–	++	++	++	–
3	X			X		1.4	–	–	+		
4	X	X	X			5.2	+	++	++		
5	X	X	X		X	95.2	+	n.d.	n.d.		
6	X	X	X	X		37.9	+	n.d.	n.d.		
7	X	X				10.5	+	n.d.	n.d.		
8	X	X	X	X		21.3	–	++	++		
9	X	X	X	X		14.4	–	n.d.	+		
10	X	X	X		X	41.0	+	++	++		–
11	X	X				4.8	–	++	++		–
12	X	X	X		X	187.7	–	+	+		
13	X	X			X	43.5	–	–	–		
14	X	X	X		X	143.3	–	–	–		
15	X	X	X		X	89.9	–	n.d.	n.d.		
16	X	X			X	133.0	–	n.d.	n.d.		
17	–	–	–		X	43.1	–	n.d.	n.d.		
18	–	–	–		X	11.0	–	–	–		
19	–	–	–		X	52.3	–	–	–		
20	–	–	–		X	21.6	–	–	–		

Clear evidence of a secondary lesion is denoted by “+”. “++” denotes especially large secondary lesions. Patients 1 through 12 showed clear evidence for delayed midbrain changes (DWI and T2) in ipsilateral substantia nigra occurring between days 6 and 10. Despite heavy motion artifacts, scans of patients 13 through 15 still showed some evidence for secondary midbrain changes (+). Several patients missed scans (n.d.). The primary reason for this was that patients had either been transferred to a rehabilitation center or discharged. Control stroke patients (patients 17 through 20) did not show evidence of secondary midbrain changes. Note that cortical involvement or the overall size of the infarct did not affect the development of secondary midbrain changes. Normalization of ADC was observed at late measurements in single patients (days 72, 90, and 144).

### Postprocessing and Data Analysis

The following definitions apply: (a) ‘Primary ischemic lesion’ means the early T2 hyperintense signal in the MCA territory. (b) The term ‘secondary MRI lesion’ is used for delayed T2/DWI signal changes that emerge in the ipsilateral midbrain after stroke. ROIs of these two lesion types were used independently in further analyses. As described in detail below, lesions were identified separately on T2 and DWI scans. However, because of higher spatial resolution and easier-to-process coronal orientation, volumetry and lesion maps were generated from T2 scans only. In general, DWI lesions matched T2 lesions well. However, because of different scan techniques, we did not calculate the degree of overlap between DWI and T2 lesions.

For coronal T2 scans obtained on day 10 (or on the latest available scan before day 10), binary masks distinguishing between the hyperintense-appearing primary and secondary lesions, (coded as “1”), and non-infarcted areas (“0”) were generated for later usage as regions of interests (ROI) and as the basis for subsequent volumetric measurements using the MRIcro software (http://www.mricro.com). Lesion segmentation was performed manually by an experienced investigator. To combine left- and right-sided infarcts into a single analysis, all images with right-hemispheric strokes were mirrored right to left.

Furthermore, coronal T2-weighted images were co-registered with coronal section outlines of a digitized atlas of the human brain [22, 23] using an in-house developed software (“THAT”). In a first step, the interhemispheric midsagittal plane and the orthogonal plane through the bicommissural line between the anterior and posterior commissures were determined as reference planes. Then the images were repetitively transformed (translation, rotation, linear scaling) by the investigator with respect to structural landmarks (such as thalamus, caudate nucleus, corticospinal tract, putamen, wall of the third ventricle) [22]. Transformations generated during the co-registration procedure were used to spatially adjust the ROIs to the stereotactic space of the digital atlas. The transformed image data as well as their corresponding ROIs were subsequently re-sliced into two different iso-voxel spaces (1 x 1 x 1 mm; 0.25 x 0.25 x 0.25 mm) using SPM8 (Statistical Parametric Mapping; Wellcome Department for Cognitive Neurology, University College London, UK) running on MATLAB 7.3 (The MathWorks, Inc.; Natick, MA, USA). Finally, the relative frequencies of the primary and secondary lesions were voxel-wisely calculated for both iso-voxel spaces and the resulting maps were subsequently color-coded. These cluster maps were overlaid on the coronal section outlines of the digital anatomic atlas to determine the lesion topography more precisely.

For the determination of changes in ADC in the secondary midbrain lesion as a function of time, the respective ROI was delineated in axial DWI images (trace-weighted) obtained on day 10 or on the latest available scan between days 6 and 10, respectively, using MRIcro. Imaging data of preceding scans were co-registered with this reference using SPM8 and the mean ADC values of the lesions were quantified for all available time points. For the determination of contralateral ADC values as an intra-subject reference value, ROIs were mirrored to the corresponding contralateral neuroanatomic structures.

To visualize the spatial distribution of the primary lesion, DWI data sets on day 10 or on the latest available scan, respectively, and their corresponding ROIs were additionally normalized to the brain template provided by the International Consortium for Brain Mapping (ICBM) using SPM8.

### Statistical Analysis

Values are presented as means ± SEM. Ipsilateral and contralateral ADC measurements were compared using paired t-tests.

## Results

Sixteen consecutive striatal stroke patients and four control stroke patients were enrolled into the study. Patients’ baseline characteristics are summarized in Table 2. The primary ischemic lesion was evaluated on day 2. Follow-up scans were performed as indicated in Table 1 using T2-weighted and DWI scans.

**Table 2:** Baseline characteristics of study participants.

	striatal stroke patients	control stroke patients
No.	16	4
Age (mean ± S.E.M.)	70.4 ± 9.7	60.3 ± 26.3
Female gender, n (%)	8 (50)	3 (75)
NIHSS score, median (25^th^ and 75^th^ percentiles)	12 (5.5; 16.3)	5 (3; 9.8)
Right side of infarction, no. (%)	7 (44)	4 (100)

**Fig 1:**
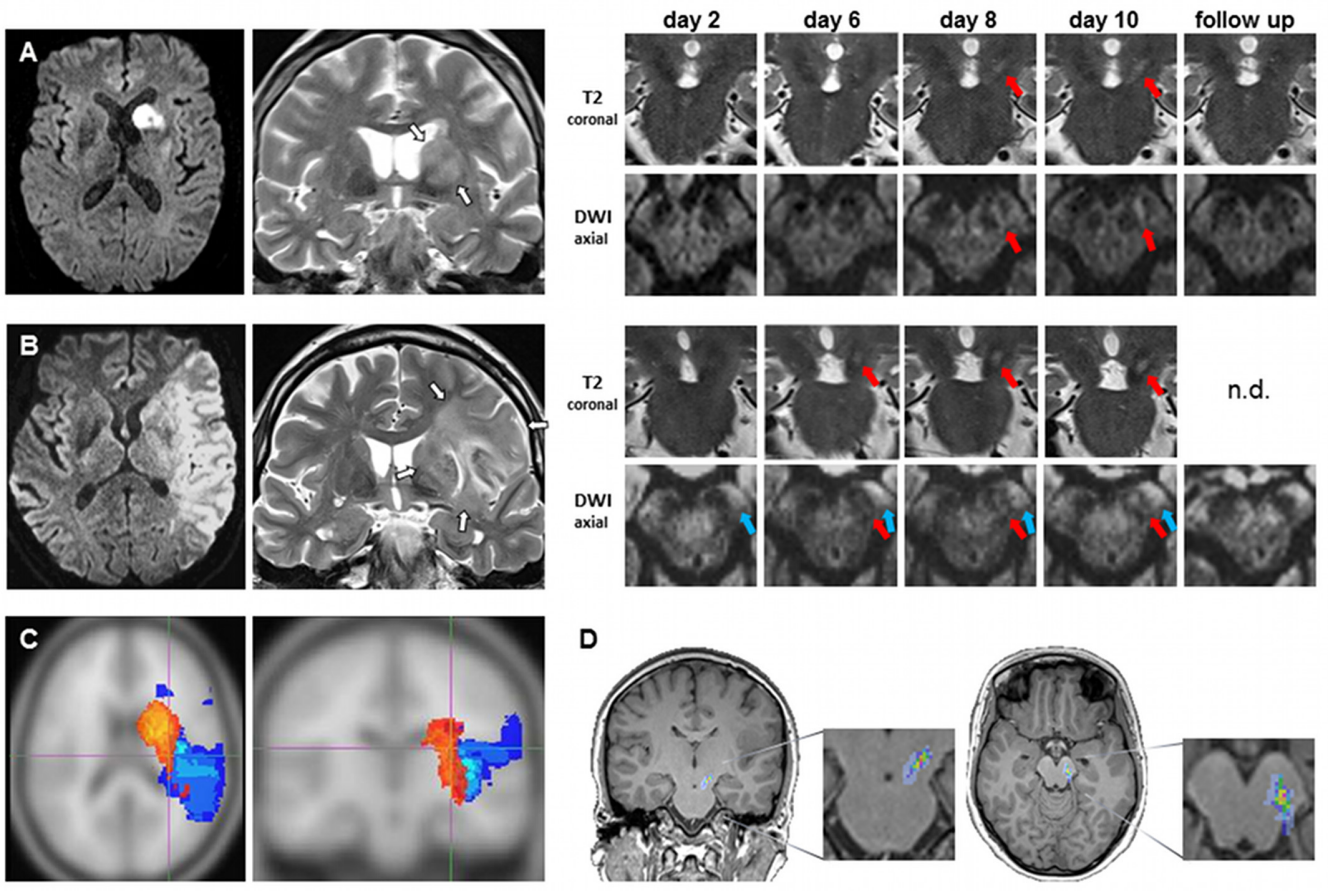
Subacute hyperintensity in ipsilateral midbrain at a delayed time point after striatal stroke. (A, B) MRI scans of two exemplary patients showing primary ischemic lesion confined to striatum (A) or involving striatum (B) in axial diffusion-weighted (DWI, left) and coronal T2-weighted (T2) imaging (white arrows; 2^nd^ from left). On the right side, coronal views through the midbrain display the development of an ipsilateral hyperintense lesion occuring between days 6 to 10 after stroke (red arrows). Note that corticospinal degeneration (blue arrows in B) associated with cortical involvement is detectable before the emergence of these secondary exofocal changes in midbrain. (C) Frequency of the anatomic distributions of the primary ischemic lesions (12 striatal stroke and 4 control stroke patients). Lesions are overlayed on the ICBM human brain template. Infarcts associated with secondary midbrain changes are coded in red, infarcts not associated with midbrain changes are coded in blue. Only frequencies of at least 25% are displayed. (D) Localization of secondary exofocal midbrain changes (n = 12 striatal stroke patients). For the purpose of this illustration, secondary lesions (day 10 or latest available scan before day 10) were adjusted to and superimposed on coronal and transverse T1-weighted images of a single patient.

Fig 1 illustrates the emergence of secondary exofocal midbrain changes. Primary ischemic infarcts (Fig 1A and 1B) involving striatal structures resulted in hyperintense ipsilateral midbrain lesions that emerged between days 6 and 10 after stroke (red arrows in Fig 1A and 1B). None of the 4 patients with MCA infarctions sparing striatal structures showed any evidence of such secondary changes through day 10 (Table 1; patients 17–20). Note that exofocal midbrain changes in gray matter have to be distinguished from hyperintense lesions in the pyramidal tract within the brain stem white matter. The latter result from Wallerian degeneration of the corticospinal tract (blue arrows in Fig 1B) and become clearly detectable from day 2 of stroke onwards.

**Fig 2:**
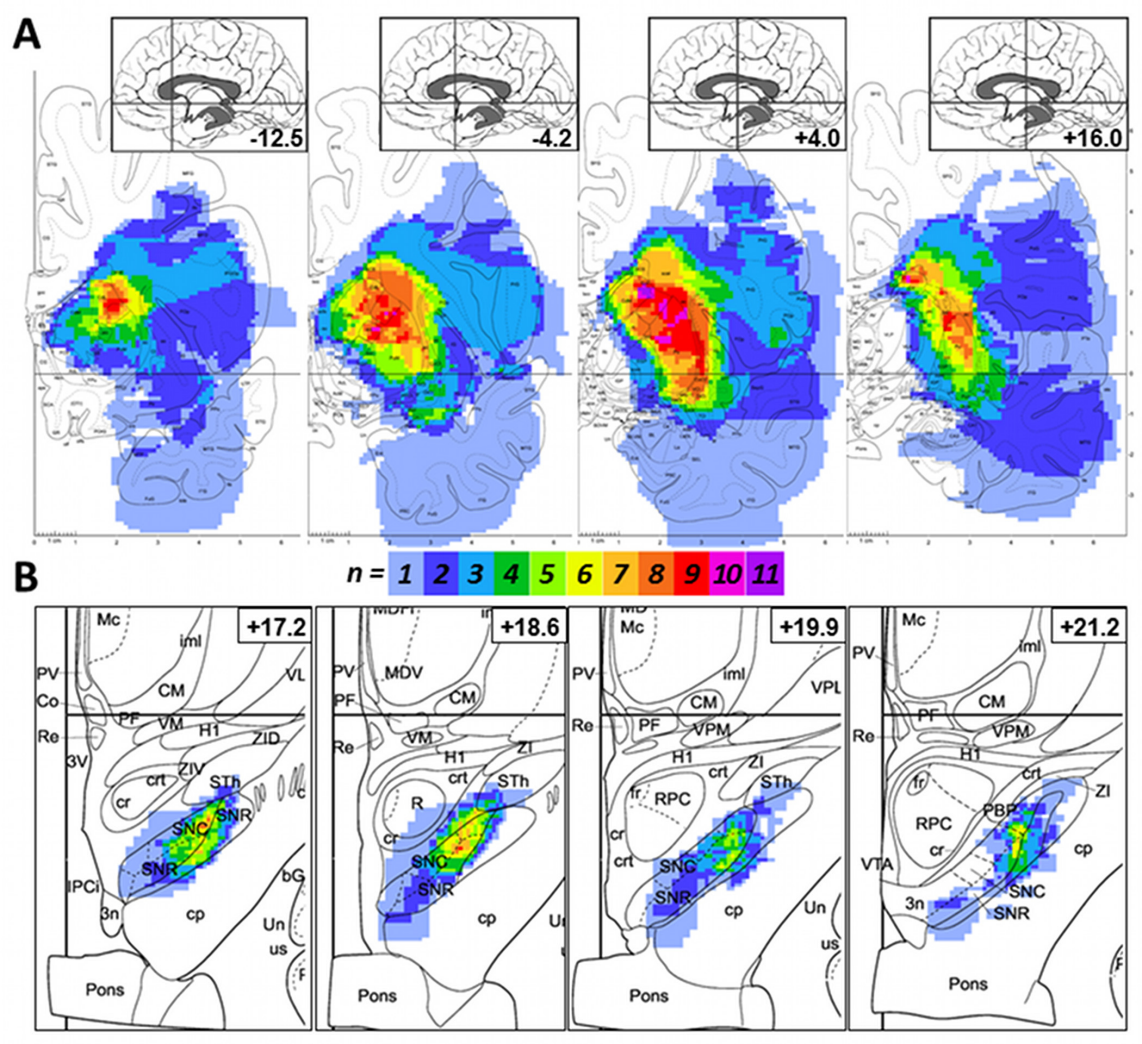
Ischemic lesions of striatum elicit secondary changes in ipsilateral substantia nigra. (A) Color coding of the relative frequency of “ischemic” voxels in the primary ischemic lesion of striatal stroke patients (n = 12) who subsequently developed secondary midbrain changes. Cd: Caudate nucleus (n_max_ = 11); Pu: putamen (n_max_ = 10). (B) Color coding of the relative frequency of voxels showing secondary changes in midbrain in these patients. SNC: substantia nigra, pars compacta (n_max_ = 9). SNR: substantia nigra, pars reticulata. Numbers ranging from -12.5 to +21.2 denote Talairach y-coordinates.

Next, we studied the anatomic distributions and relative frequencies of the primary ischemic lesions in relation to the emergence of secondary midbrain changes (Table 1; Figs 1C and 2A). Infarcts associated with delayed exofocal midbrain changes (red color-coded in Fig 1C) consistently involved striatal structures (caudate nucleus, putamen; Fig 2A). Similarly, we studied the frequencies of the precise anatomic localizations of secondary exofocal lesions (Figs 1D and 2B). Co-registration with a digitized anatomic atlas [22, 23] showed that the core area of the secondary exofocal midbrain lesions was consistently located in the pars compacta of the substantia nigra.

**Fig 3:**
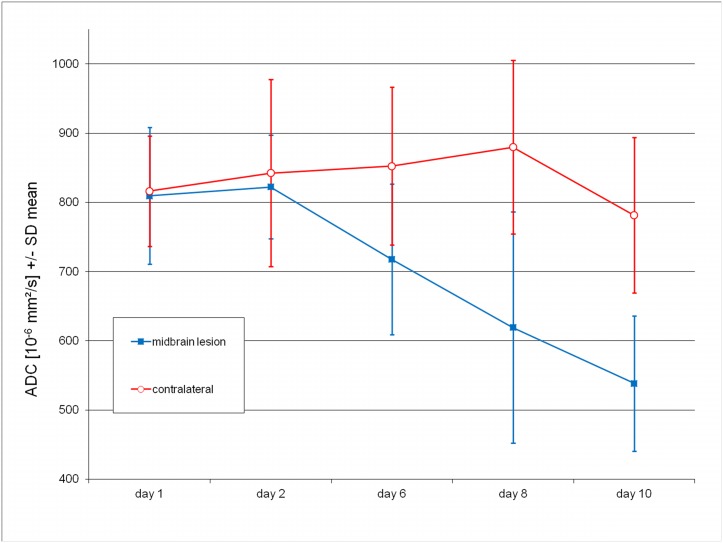
Decline in ADC values in ipsilateral midbrain after striatal stroke. ADC values in the exofocal midbrain lesion (blue squares) showed a significant delayed decrease relative to the corresponding nonlesioned (red circles) contralateral midbrain area (paired t-tests; day 6: p<0.02 [n = 12], day 8: p<0.005 [n = 9], day 10: p<0.0005 [n = 7]).

Finally, we measured ADC values of the ipsilateral secondary exofocal lesions over time. As compared to the corresponding midbrain area of the contralateral hemisphere, ADC values in the ipsilateral lesion showed a delayed sharp decline through day 10 (Fig 3).

## Discussion

This study has the following major findings: Ischemic lesions in the striatum lead to secondary changes in ipsilateral midbrain gray matter. Co-registration of high-resolution images at 3 T with a neuroanatomic atlas revealed that in human stroke patients these secondary midbrain changes emerge particularly in the substantia nigra pars compacta. As compared to hyperintense lesions in the pyramidal tract, which are caused by Wallerian degeneration of the corticospinal tract, secondary midbrain hyperintensities were not present on the initial MRI scans but appeared in a delayed fashion between days 6 and 10 after striatal stroke.

Cranial MRI provides an excellent anatomic definition of the location of the primary ischemic lesion and can also be used in the subacute and chronic phases after stroke. It is therefore important that clinicians be aware of the delayed MRI changes described here so as not to confuse them with a second stroke. As compared to an earlier study of striatal stroke patients at 0.5 T, which reported the first occurrence of a T2 hyperintensity in the midbrain, on average, at more than 14 days after stroke onset [16], our study at higher field strengths revealed that secondary changes in the substantia nigra are already underway during the first week after stroke. The combination of a T2 hyperintensity and decreased ADC values suggests both cytotoxic edema as well as vasogenic edema[24–26]. A similar pattern of MRI changes in midbrain as described here for human stroke patients with striatal involvement has previously been reported after occlusion of the middle cerebral artery in rats [12]. Conceivably, such secondary changes may emerge as a novel target for neuroprotection with an extended time window [8, 27]. Importantly, in an experimental study in mice subjected to transient occlusion of the MCA, the exofoxal T2 hyperintensity in the midbrain was associated with activation of microglia and clearly preceded overt neuron loss [8].

While experimental stroke studies in rats mainly point to involvement of the pars reticulata of the substantia nigra, our study, as well as an earlier MRI study in human patients [16], found that secondary changes were especially prominent in, albeit not limited to, the pars compacta (Fig 2B). Several explanations have been offered for this apparent discrepancy, including differences in age, species, or the exact location of the primary ischemic lesion in the striatum [16]. The striatonigral pathway predominantly projects to the pars reticulata of the ipsilateral substantia nigra [28]. Destruction of this inhibitory GABAergic pathway after focal stroke may result in disinhibition, and ultimately, delayed transneuronal (i.e., transsynaptic) degeneration, primarily of neurons in the pars reticulata of the substantia nigra [2, 29]. By contrast, disruption of the nigrostriatal pathway may result in retrograde degeneration of dopaminergic (i.e. tyrosine hydroxylase-expressing) neurons located primarily in the substantia nigra pars compacta [30]. It is likely that both of these mechanisms also come into play in human striatal stroke patients, depending on the precise neuroanatomic location of the primary striatal lesion.

What might be the clinical relevance of delayed exofocal neurodegeneration? In the current study, no apparent clinical deterioration was observed with the onset of secondary midbrain MRI changes. However, using a middle cerebral artery occlusion model in the mouse, we previously demonstrated that antidepressants prevent delayed neurodegeneration in the midbrain and thereby attenuate the depression-like behavioral syndrome that typically evolves in the subacute stages of recovery [31]. We speculate that, in stroke survivors, secondary neuronal loss typically manifests in a delayed fashion in the form of subtle cognitive and neuropsychiatric deficits [32], which may negatively impact long-term functional outcomes. Conceivably, exofocal neuronal loss may also decrease a patient’s ‘cognitive reserve’, rendering him or her more vulnerable to behavioral effects of subsequent brain pathology [32].

In conclusion, exofocal post-ischemic neuronal degeneration in the ipsilateral substantia nigra depends on stroke topography and becomes detectable by MRI between days 6 and 10 after stroke. DWI and T2-weighted imaging at 3 T offer reliable detection of these secondary midbrain changes. The further study of these delayed processes of lesion progression after stroke may aid research in developing new strategies aimed at preventing secondary neuron loss.

A potential caveat concerning our data is the relatively small size of the control group. Although the current study focusses primarily on striatal infarctions as related to secondary lesion development, which is only present in this group, the small sample size of the ‘non-striatal’ control group and differences in baseline patient characteristics (e.g. NIHSS scores; Table 2) may limit the generalizability of our findings.

## Supporting Information

S1 Table Individual ADC Values of EPND compared to contralateral side (please refer to Fig 3). ADC Values (ADC [10^−6^ mm^2^/s +/- SD mean) of EPND (ROIs in axial DWI (ADC)) in 12 patients ipsilateral to primary lesion (upper panel) compared to mirrored controlateral ROIs (bottom panel). As mentioned in the manuscript, late measurements (days 12, 72 and 144) were done in single patients and show ADC normalization on day 72 and 144. P-values for the comparison of ipsilateral and contralateral ADC values were calculated using Students paired T-tests.
(PPTX)
