# Supplementary material for: Striatal Infarction Elicits Secondary Extrafocal MRI Changes in Ipsilateral Substantia Nigra
Source: PLoS One. 2015 Sep 1;10(9):e0136483. doi: 10.1371/journal.pone.0136483 (PMC4556671; doi:10.1371/journal.pone.0136483)
Supplement: S1 Table — ADC Values (ADC [10−6 mm2/s +/- SD mean) of EPND (ROIs in axial DWI (ADC)) in 12 patients ipsilateral to primary lesion (upper panel) compared to mirrored controlateral ROIs (bottom panel). As mentioned in the manuscript, late measurements (days 12, 72 and 144) were done in single patients and show ADC normalization on day 72 and 144. P-values for the comparison of ipsilateral and contralateral ADC values were calculated using Students paired T-tests. (PPTX) [file pone.0136483.s001.pptx]

## Slide 1
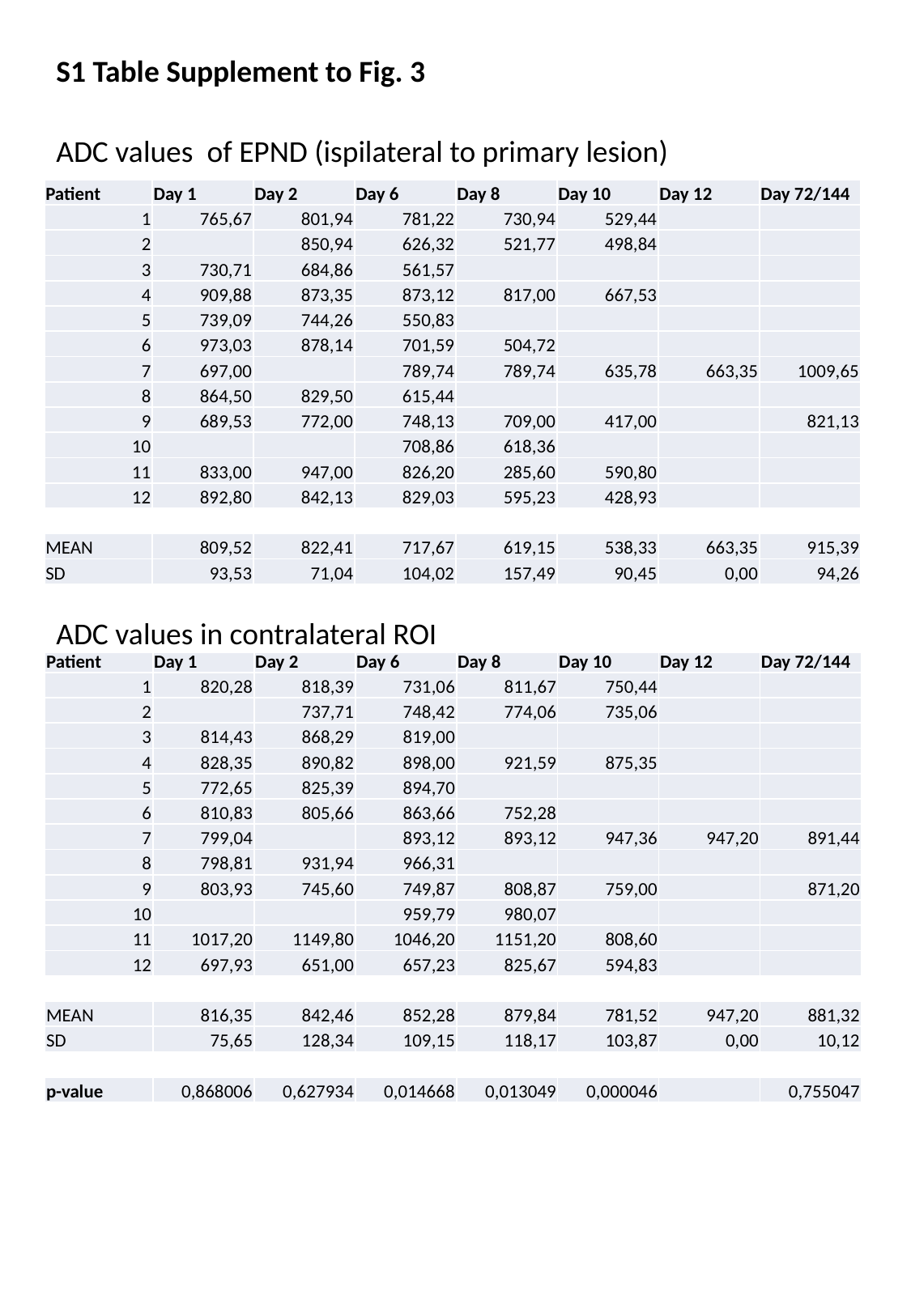

S1 Table Supplement to Fig. 3
ADC values of EPND (ispilateral to primary lesion)
| Patient | Day 1 | Day 2 | Day 6 | Day 8 | Day 10 | Day 12 | Day 72/144 |
| --- | --- | --- | --- | --- | --- | --- | --- |
| 1 | 765,67 | 801,94 | 781,22 | 730,94 | 529,44 | | |
| 2 | | 850,94 | 626,32 | 521,77 | 498,84 | | |
| 3 | 730,71 | 684,86 | 561,57 | | | | |
| 4 | 909,88 | 873,35 | 873,12 | 817,00 | 667,53 | | |
| 5 | 739,09 | 744,26 | 550,83 | | | | |
| 6 | 973,03 | 878,14 | 701,59 | 504,72 | | | |
| 7 | 697,00 | | 789,74 | 789,74 | 635,78 | 663,35 | 1009,65 |
| 8 | 864,50 | 829,50 | 615,44 | | | | |
| 9 | 689,53 | 772,00 | 748,13 | 709,00 | 417,00 | | 821,13 |
| 10 | | | 708,86 | 618,36 | | | |
| 11 | 833,00 | 947,00 | 826,20 | 285,60 | 590,80 | | |
| 12 | 892,80 | 842,13 | 829,03 | 595,23 | 428,93 | | |
| | 10 | 10 | 12 | 9 | 7 | 1 | 2 |
| MEAN | 809,52 | 822,41 | 717,67 | 619,15 | 538,33 | 663,35 | 915,39 |
| SD | 93,53 | 71,04 | 104,02 | 157,49 | 90,45 | 0,00 | 94,26 |
ADC values in contralateral ROI
| Patient | Day 1 | Day 2 | Day 6 | Day 8 | Day 10 | Day 12 | Day 72/144 |
| --- | --- | --- | --- | --- | --- | --- | --- |
| 1 | 820,28 | 818,39 | 731,06 | 811,67 | 750,44 | | |
| 2 | | 737,71 | 748,42 | 774,06 | 735,06 | | |
| 3 | 814,43 | 868,29 | 819,00 | | | | |
| 4 | 828,35 | 890,82 | 898,00 | 921,59 | 875,35 | | |
| 5 | 772,65 | 825,39 | 894,70 | | | | |
| 6 | 810,83 | 805,66 | 863,66 | 752,28 | | | |
| 7 | 799,04 | | 893,12 | 893,12 | 947,36 | 947,20 | 891,44 |
| 8 | 798,81 | 931,94 | 966,31 | | | | |
| 9 | 803,93 | 745,60 | 749,87 | 808,87 | 759,00 | | 871,20 |
| 10 | | | 959,79 | 980,07 | | | |
| 11 | 1017,20 | 1149,80 | 1046,20 | 1151,20 | 808,60 | | |
| 12 | 697,93 | 651,00 | 657,23 | 825,67 | 594,83 | | |
| N | 10 | 10 | 12 | 9 | 7 | 1 | 2 |
| MEAN | 816,35 | 842,46 | 852,28 | 879,84 | 781,52 | 947,20 | 881,32 |
| SD | 75,65 | 128,34 | 109,15 | 118,17 | 103,87 | 0,00 | 10,12 |
| | | | | | | | |
| p-value | 0,868006 | 0,627934 | 0,014668 | 0,013049 | 0,000046 | | 0,755047 |
